# Effect of sidt2 Gene on Cell Insulin Resistance and Its Molecular Mechanism

**DOI:** 10.1155/2020/4217607

**Published:** 2020-09-11

**Authors:** Qian-Ying Xiong, Chao-Qun Xiong, Li-Zhuo Wang, Jia-Lin Gao

**Affiliations:** ^1^Department of Endocrinology and Genetic Metabolism, Yijishan Hospital of Wannan Medical College, Wuhu 241002, China; ^2^Anhui Province Key Laboratory of Biological Macro-Molecules Research (Wannan Medical College), Wuhu 242001, China; ^3^Department of Biochemistry and Molecular Biology, Wannan Medical Collage, Wuhu 241001, China

## Abstract

**Background:**

Sidt2 (SID1 transmembrane family, member 2) is a multiple transmembrane lysosomal membrane protein newly discovered in our previous study. In the previous study, we used gene targeting technique to make a mouse model of sidt2 gene knockout (sidt2^−/−^). It was found that sidt2^−/−^ mice showed elevated fasting blood glucose and impaired glucose tolerance, showing a disorder of glucose metabolism, suggesting that sidt2 may be closely related to insulin resistance. We used 3T3-L1 adipocytes, C2-C12 myoblasts, and HEPA1-6 hepatoma cells as subjects to observe the effects of sidt2 on insulin-stimulated glucose uptake and the abovementioned insulin signal transduction pathways, and then to explore the effect of sidt2 on peripheral tissue insulin resistance and its possible molecular mechanism.

**Methods:**

(1) Lentiviruses with sidt2 gene knockout and puromycin resistance were constructed by Crispr/cas9 vector and transfected into 3T3-L1 adipocytes, C2-C12 myoblasts, and HEPA1-6 hepatoma cells to construct sidt2 knockout cell line model. (2) Glucose uptake of 3T3-L1 adipocytes, C2-C12 myoblasts, and HEPA1-6 hepatoma cells stimulated by insulin was detected by glucose detection kit, and the results were analyzed. (3) Sidt2 knockout group and control group of 3T3-L1 adipocytes, C2-C12 myoblast, and HEPA1-6 hepatoma cells were cultured according to the routine method. The total proteins of the above cells were extracted, and the expression of PAKT (thr308), PI3-K, and PIRS-1 (ser307) in the IRS-1 signaling pathway of the three groups was detected by western blot technique.

**Results:**

(1) The sidt2 elimination models of 3T3-L1 adipocytes, C2-C12 myoblasts, and HEPA1-6 hepatoma cells were successfully constructed. (2) It was found that the glucose uptake of cells in the sidt2 knockout group was lower than that in normal group under insulin stimulation through the detection of glucose concentration in the cell culture medium. (3) It was found that the expression of PAKT (thr308) and PI3-K protein decreased and the expression of PIRS-1 (ser307) protein increased in sidt2^−/−^ group compared to the control group.

**Conclusions:**

sidt2 knockout can reduce glucose uptake in peripheral tissue under insulin stimulation, which may lead to peripheral tissue insulin resistance by affecting the IRS-1 signal pathway.

## 1. Introduction

Insulin resistance (Insulin Resistance, IR) refers to the decreased sensitivity of insulin target tissue (adipose tissue, skeletal muscle, liver) to insulin. In the early stage, islet *β*-cells can compensatively increase insulin secretion to make up for its deficiency, but over time, the function of islet *β*-cells will gradually fail, leading to abnormal glucose tolerance and the occurrence of diabetes [1]. IR can coexist with central obesity, hypertension, and dyslipidemia, which is called metabolic syndrome, which is a serious threat to people's life and health [2]. At present, it is a hot topic to find out the factors related to insulin resistance, to explore the mechanism of its influence on insulin resistance, and to provide new molecular targets for the prevention and treatment of type 2 diabetes mellitus (T2DM).

The insulin signal pathway is closely related to the occurrence of insulin resistance. Changes in the activity of key signal molecules in the insulin signal pathway will affect the transmission of insulin signal, and then affect the biological activity of insulin, thus affecting the occurrence of insulin resistance and T2DM. A large number of studies have confirmed that there are two main pathways of insulin postreceptor signal transduction: one is the IRS-1-PI3-K-Akt pathway; the other is RAS mitogen-activated protein kinase (mitogen-activated protein kinase, MAPK) pathway. MAPK pathway is mainly related to gene transcriptional regulation, and insulin mainly mediates its metabolic regulation through the PI3-K pathway. As the main signal pathway of insulin, IRS-1-PI3-K-Akt pathway is involved in the physiological mechanism of glucose metabolism in vivo [3–5]. IRS-1 is a pivotal molecule in the insulin signal transduction pathway, which mainly transduces and amplifies insulin signal. IRS-1 contains 22 potential tyrosine phosphorylation sites and more than 40 potential serine-threonine phosphorylation sites. Without activation, serine on IRS-1 is phosphorylated, but tyrosine phosphorylation is enhanced after insulin activation. When IRS-1 is activated by insulin receptor tyrosine kinase, more than 8 tyrosine are phosphorylated and can bind to PI3-K protein containing the SH2 domain [6]. PI3-K is a lipid kinase that plays a key role in mediating insulin metabolism. PI3-K consists of a regulatory subunit (p85) with molecular weight 85kD and a catalytic subunit (p110) of 110 kD. P85 inhibits p110 in resting state. Under insulin stimulation, IRS binds to p85, the inhibition is relieved, and p110 is activated. After PI3-K activation, downstream PKB/Akt is activated by PKD phosphorylation. Akt, also known as protein kinase B (PKB), is a downstream signal molecule of PI3-K. PI3-K can activate PKB/Akt by phosphorylating threonine and serine at position 308 and 473, and promote downstream glucose transporter translocation to the cell surface, resulting in a corresponding increase in glucose uptake [7].

At present, more than 50 kinds of lysosomal membrane proteins have been found [8–10], but the exploration of the lysosome and its membrane proteins has been unpopular, and the research is mainly focused on the field of autophagy and lysosomal storage disease. Although the specific mechanism of T2DM has been the focus of research at home and abroad, it is also mainly focused on the signal regulation of insulin secretion and secretion [11–13]. However, there are few studies on the relationship between lysosomal membrane proteins and insulin resistance. Sidt2 (SID1 transmembrane family, member 2) is a multiple transmembrane lysosomal membrane protein found recently in our previous study. Mouse sidt2 gene is located on chromosome 9A5.2 and consists of 26 exons, mainly encoding 832 amino acid residues [14]. In the previous study, we used gene targeting technology to make sidt2 gene knockout (sidt2^−/−^) mouse model and found that Sidt2^−/−^ mice showed elevated fasting blood glucose, impaired glucose tolerance, and disorder of glucose metabolism, suggesting that sidt2 may be closely related to insulin resistance [15]. In this study, we will study the effects of sidt2 on insulin-stimulated glucose uptake in 3T3-L1 adipocytes, C2-C12 myoblasts, and HEPA1-6 hepatoma cells, and on IRS-1-PI3-K-Akt signaling pathway, in order to reveal the effect of sidt2 on cellular insulin resistance and its possible molecular mechanism, and provide a new direction for the diagnosis and treatment of type 2 diabetes.

## 2. Methods

### 2.1. Cell Culture and Establishment of sidt2 Knockout Cell Model

3T3-L1 adipocytes were purchased from the Cell Resource Center of Shanghai Academy of Life Sciences, Chinese Academy of Sciences, while C2-C12 myoblasts and HEPA1-6 hepatoma cells were purchased from ATCC cell bank. 3T3-L1 adipocytes, C2-C12 myoblasts, and HEPA1-6 hepatoma cells were cultured in a DMEM medium containing 10%FBS in an incubator at 37°C and 5% CO2. When the cells grew to the logarithmic phase and the density was 80%, they could be used in the experiment. The above three kinds of cells were transferred to a six-well plate and cultured to about 60% of the cell density, and the lentivirus prepared in advance for knocking out sidt2 and having puro resistance was added to the culture medium. In order to make the lentivirus better-transfected cells, the cells were not treated within 48 hours, and the culture medium could be replaced after 48 hours. 72 hours after the virus was added, the new medium was replaced, and an appropriate amount of puro was added to it to screen cells (the amount of puros varied according to different cell lines). At the same time, it was necessary to add polybrene, to increase the transfection efficiency of the virus. The cells were screened by adding puro for three consecutive days, and the screened cells were cultured normally.

### 2.2. Glucose Uptake

The normal cultured cells were subcultured in a six-well plate, and the cells were in good condition with a density of about 60%. The cells were incubated with 10^−3^ mmol/l insulin for 15 minutes, and then replaced with a proper amount of culture solution and preconfigured glucose solution, so that each well cell in the six-well plate was placed in a medium with different glucose concentrations (HEPA1-6 cells, 3T3-L1 cells: 5.5, 36.1, 50, 70, 85, 125 mmol/l, C2-C12 cells: 5.5, 11.5, 25, 45, 60, 100 mmol/l). After being incubated in the incubator of 37°C and 5%CO2 for 24 hours, the liquid transfer gun absorbed the culture solution from each hole and put it into the 1.5 ml EP tube. According to the instructions of the glucose detection kit (Nanjing Jiancheng Institute of Biological Engineering), the concentration of glucose in different holes was detected and analyzed.

### 2.3. Western Blotting

The total protein was extracted with high-efficiency RIPA cell lysate (Solarbio, China), and the concentration of total protein was determined by Nanodrop2000 ultramicrospectrophotometer. Total cell lysates were separated on 10% SDS-PAGE and blotted with the following primary antibodies: rabbit anti-*β*-actin (1 : 5000, CST, USA), rabbit anti-PAKT (thr308) (1 : 1000, CST, USA), rabbit anti-PI3-K (P85) (1 : 1000, CST, USA), and rabbit anti-PIRS-1 (1 : 1000, CST, USA) and rabbit anti-PIRS-1 (1 : 1000, CST, USA).

### 2.4. Statistical Analysis

Data were presented as the means ± SEM of at least three independent experiments. Unpaired *t*-test was used to assess the statistical comparison between the two groups. GraphPad Prism 8.0 software was used for statistical analyses. Values of *P* < 0.05 were considered significant differences.

## 3. Results

### 3.1. Successful Construction of sidt2 Gene Knockout Models in Three Cell Lines

**Figure 1:**
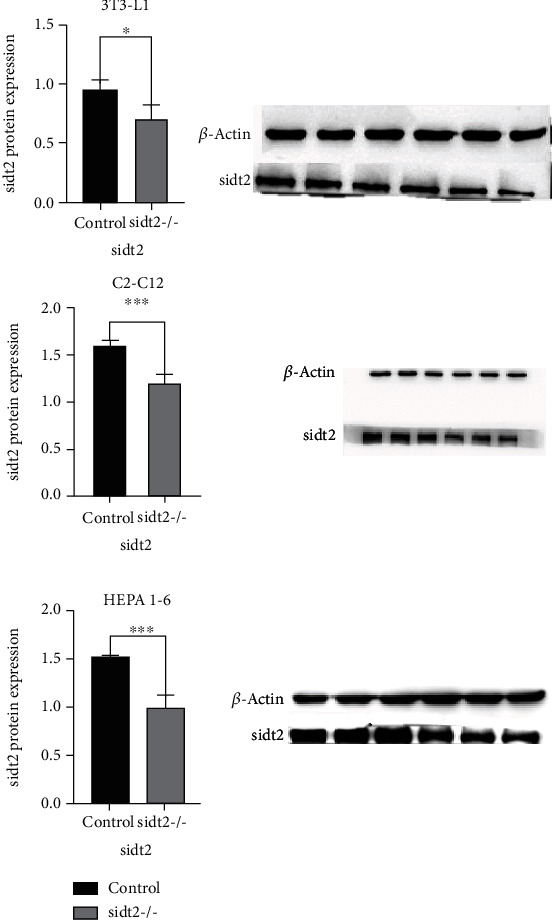
WB results of three cell lines, the leftmost three holes are the control group, and the right three holes are the sidt2 elimination group (^∗^: *P* < 0.05, ^∗∗^: *P* < 0.03, ^∗∗∗^: *P* < 0.01). The statistical chart shows that there is a significant difference in the expression of sidt2 protein between the control group and the elimination group.

The puromycin-resistant cell lines infected by the virus were selected according to the above methods for culture, and the cell proteins were extracted and verified by Western blotting and analyzed (as shown in Figure 1). The expression of Sidt2 protein in the sidt2 elimination group of the three cell lines was significantly lower than that in the control group (*P* < 0.05), which basically proved that the model was established successfully.

### 3.2. Effect of Sidt2 on Glucose Uptake of Cells

**Figure 2:**
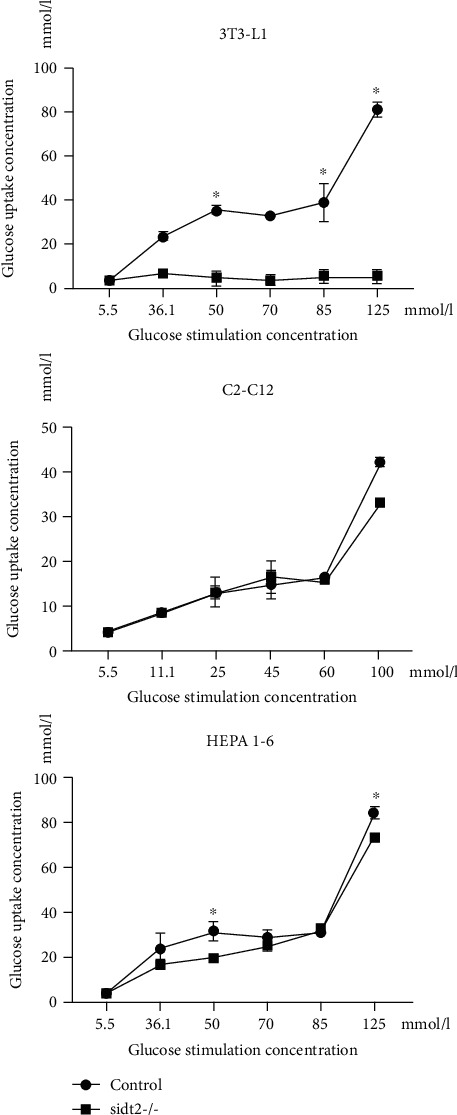
Glucose uptake of three cell lines (^∗^: *P* < 0.05). Glucose uptake in the sidt2 knockout group was lower than that in the control group, and the difference was significant at some concentrations.

We use the above cell culture methods to culture cells and induce them to differentiate and mature. The glucose solution was added to the cell culture medium to change the glucose concentration of the living environment. 24 hours later, the glucose detection kit was used to detect the remaining glucose concentration in the culture solution, so as to calculate and evaluate the glucose uptake of the cells. As shown in Figure 2, the sidt2 gene promotes glucose uptake under insulin stimulation in three cell lines.

### 3.3. Effect of Sidt2 on the Expression of Key Proteins in IRS-1 Signal Pathway

**Figure 3:**
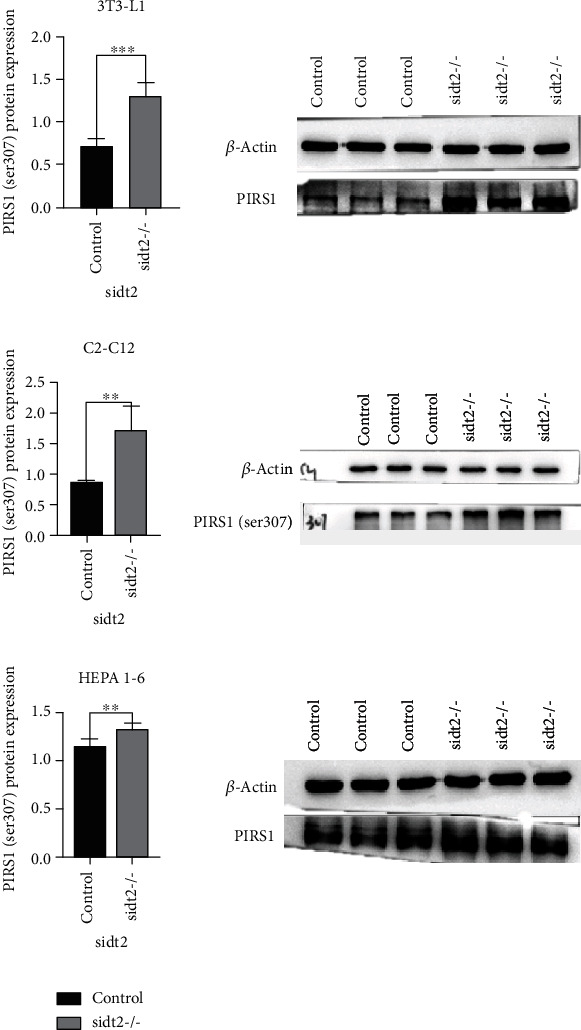
WB results of three cell lines (^∗^: *P* < 0.05, ^∗∗^: *P* < 0.03, ^∗∗∗^: *P* < 0.01). The statistical chart shows that the expression of PIRS1 (ser307) in the sidt2 knockout group is higher than that in the control group, and the difference is significant.

The phosphorylation of insulin receptor substrate-1 serine 307 (insulin receptor substrate-1 Ser307, IRS-1 Ser307) plays an important negative role in insulin signal transduction. After IRS-1Ser307 phosphorylation, the interaction between IRS-1 and insulin receptor (insulin receptor, InsR) is weakened, and the level of tyrosine phosphorylation of IRS-1 under insulin stimulation is decreased, which further affects the downstream transduction of insulin signal. In the three cell lines, the expression of PIRS-1 (ser307) phosphorylated protein in the sidt2 knockout group was significantly higher than that in the control group, as shown in Figure 3.

**Figure 4:**
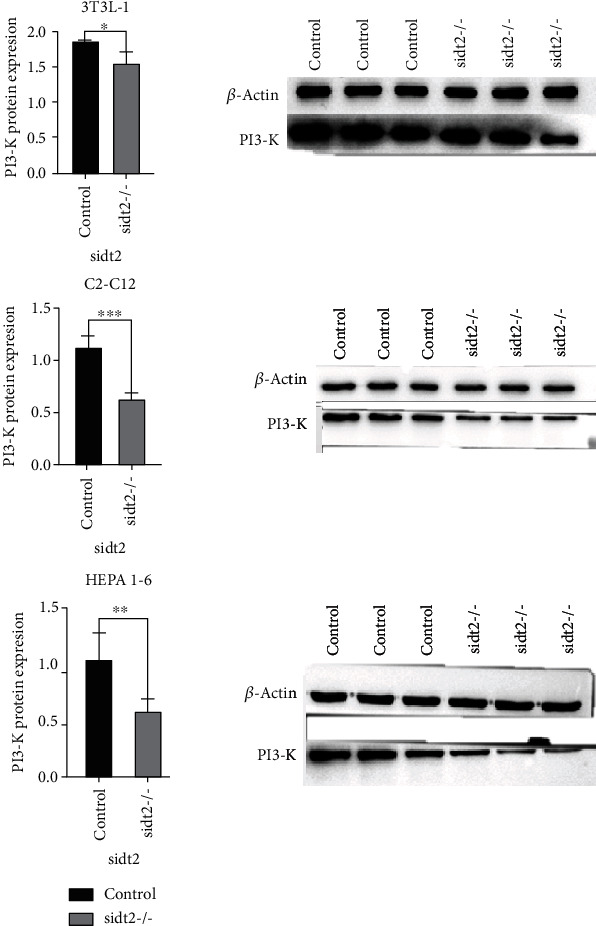
WB results of three cell lines (^∗^: *P* < 0.05, ^∗∗^: *P* < 0.03, ^∗∗∗^: *P* < 0.01). The statistical chart shows that the expression of PI3-K (P85) in the sidt2 knockout group is lower than that in the control group, and the difference is significant.

The P85 regulatory subunit of phosphatidylinositol-3 kinase (phosphatidylinositol3-kinase, PI3K) plays an important role in activating the activity of PI3K. As shown in Figure 4, the expression of PI3-K (P85) protein in the sidt2 knockout group was significantly lower than that in the control group.

**Figure 5:**
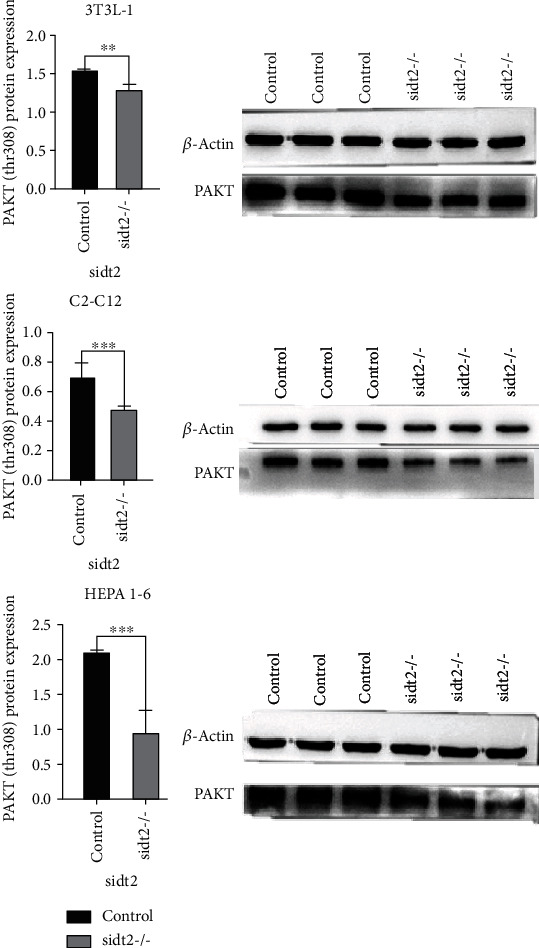
WB results of three cell lines (^∗^: *P* < 0.05, ^∗∗^: *P* < 0.03, ^∗∗∗^: *P* < 0.01). The statistical chart shows that the expression of PAKT (thr308) in the sidt2 knockout group is lower than that in the control group, and the difference is significant.

AKT is the downstream gene of the IRS-1 signal pathway and the target gene of PI3-K. It has two phosphorylation sites, ser473 and thr308. Phosphorylation can promote the transfer of GLUT-4 from the capsule to the cytoplasm and promote glucose uptake and utilization. Activation of AKT phosphorylation sites can prevent or reduce insulin resistance in cells. As shown in Figure 5, the expression of PAKT (thr308) protein in the sidt2 knockout group was significantly lower than that in the control group.

## 4. Discussion

The occurrence of T2DM is a multicause, multistep complex process, and its pathogenesis is also affected by many factors. One of the most important pathophysiological changes is the insulin target tissue (mainly liver, muscle) producing insulin resistance or accompanied by insufficient insulin secretion. Therefore, maintaining the normal function of islet *β* cells in patients with diabetes is of great significance for improving their diabetic symptoms and treating their related symptoms. The lysosome is a kind of monolayer organelles with various forms containing a variety of hydrolases that can decompose proteins, nucleic acids, polysaccharides, and other biological macromolecules. We know that lysosome is the recycling station of intracellular metabolic waste, which is closely related to intracellular substance transport, glucose and lipid metabolism, cell membrane repair, and autophagy. With the deepening of research, we gradually understand that lysosomes also play an important role in intracellular nutrient-related signal transduction and are an important regulatory target. Insulin is synthesized and secreted by islet *β* cells and stored in the secretory vesicles of islet *β* cells. In the process of formation, the secretory vesicles of islet *β* cells are similar to lysosomes. Therefore, whether the new lysosomal membrane protein sidt2 found in previous studies has a certain regulatory effect on the insulin signal transduction pathway and insulin resistance has become our research goal.

The main physiological function of insulin is to regulate the uptake and utilization of glucose by the target tissue. Measuring the glucose uptake rate of adipocytes, myoblasts, and hepatocellular carcinoma cells stimulated by insulin in vitro is an effective method to evaluate insulin sensitivity. In this study, we found that 3T3-L1 adipocytes, C2-C12 myoblasts, and HEPA1-6 hepatoma cells all showed decreased glucose uptake stimulated by insulin after deletion of sidt2 gene, suggesting that sidt2 gene is involved in the occurrence of insulin resistance. What is the specific mechanism? From the point of view of the insulin signal transduction pathway, the obstacle of the IRS signal pathway is an important pathway of insulin resistance. Serine phosphorylation of IRS-1 is considered to be a negative regulator of the IRS-1 signaling pathway. Phosphorylation of serine 307 of IRS-1 inhibits tyrosine phosphorylation of IRS-1 and leads to insulin resistance. In this study, the sidt2 gene promotes the expression of PIRS-1 (ser307), which is consistent with the decrease of insulin-stimulated glucose uptake caused by the deletion of the sidt2 gene [16]. Akt is a downstream gene of PI3-K. PI3-K plays a key role in mediating glucose uptake. Activation of PI3-K promotes the phosphorylation of 308 threonine and 473rd serine of Akt, which mediates insulin-stimulated translocation of GLUT-4 from cytoplasm to cell membrane [16, 17]. After the deletion of the sidt2 gene, the phosphorylation activation of PI3-K and AKT decreased, but the phosphorylated IRS increased, indicating that the sidt2 gene inhibited the phosphorylation of IRS and promoted the activation of PI3-K and AKT, thus inhibiting insulin resistance.

In conclusion, we found that sidt2 gene knockout can cause insulin resistance in peripheral tissue, which may be achieved by affecting the expression of key proteins in the insulin signal transduction pathway. In future experimental studies, we can overexpress the sidt2 gene, and then observe whether insulin resistance is corrected and how the expression of key proteins in the insulin signal transduction pathway will change. In addition, whether the sidt2 gene is involved in other mechanisms of insulin resistance needs to be further studied.

## Data Availability

Data is available.
